# Editorial: Hormonal and Neuroendocrine Regulation of Energy Balance

**DOI:** 10.3389/fphys.2015.00403

**Published:** 2016-01-05

**Authors:** Maria Moreno, Antonia Lanni

**Affiliations:** ^1^Dipartimento di Scienze e Tecnologie, University of SannioBenevento, Italy; ^2^Dipartimento di Scienze e Tecnologie Ambientali, Biologiche e Farmaceutiche, Second University of NaplesCaserta, Italy

**Keywords:** energy balance, thyroid hormones, mitochondria, apelin, brown adipose tissue, melanocortin, catch up fat, uncoupling

Energy balance is the relationship between energy intake and energy expenditure plus body energy storage. In mammals, the regulation of energy balance and body weight is a complex process wherein hormonal and neuroendocrine systems cooperate via cross-talk between central and peripheral signals. When energy intake exceeds energy expenditure, an increase in body mass, of which about 70% is body fat, occurs. The maintenance of this situation, over a given period of time, results in serious disturbances such as obesity and its related metabolic disorders. Quite recently, it is emerged that also the adaptation to a deficit in energy intake promotes hyperinsulemic states and the associated risks for later development of the metabolic syndrome by increasing the efficiency of energy utilization and storage as body's adipose tissue, a phenomenon known as catch-up fat. Thus, changing the food environment can push adiposity of a population upwards and result in increased prevalence of metabolic disorders. At cellular level, mitochondria have a fundamental role in regulating metabolic pathways, and tight control of mitochondrial functions and dynamics is critical to maintaining adequate energy balance. Consequently, it is no wonder that mitochondrial dysfunctions are implicated in metabolic disorders. Recent studies have highlighted the role of: (i) hypothalamic melanocortin system as an integrative center in the regulation of energy balance and (ii) adipokines, thyroid hormones (THs) and thyroid hormone-analogs as modulators of metabolic efficiency. A better understanding of physiological processes involved in the regulation of energy balance may well offer clues for developing strategies to target metabolic disorders. For this Research Topic, our initial aim was to give a comprehensive and integrate view of the factors involved in the endocrine and neuroendocrine signaling of energy balance regulation. We expected to highlight their involvement into physiological processes and regulatory systems as well as their perturbation during pathological processes. In this endeavor, 14 papers, including 1 Opinion Article, 11 review articles, and 2 original articles reveal an appealing, broad scope of subjects involving the aim of the Topic that exploit a variety of novel informations and potential applications. Insights into highly investigated areas, concerning new avenues for regulation of energy balance by TH-derivatives such as 3,5-diiodothyronine (3,5-T2) and 3-iodothyronamine (T1AM) and analogs like GC-1, are presented in a series of review articles which describe their involvement in the regulation of lipid and energy metabolism (Senese et al.), the role of 3,5-T2 on energy balance (Goglia) and the metabolic and neurological actions of T1AM (Zucchi et al.). These contributions highlight the huge progress made in identifying “novel” mechanisms of action of TH and their derivatives, opening new perspectives in the analysis of hormonal regulation and providing new targets for potential therapeutic interventions in metabolic and endocrine diseases. A review contribution by Silvestri et al. highlights the important role of proteomic approaches in the understanding of complex mechanisms such as those involved in thyroid control of metabolism. The authors of this contribution discuss new leads (i.e., target proteins and metabolic pathways) emerging in applying proteomics to the actions of 3,5-T2 and Triiodothyronine (T3) in conditions of altered energy metabolism in animal tissues having a central role in the control of energy balance. A relatively new area of research is covered by Kim et al. who review the role of the melanocortin system in the control of energy homeostasis. Many peripheral signals including hormones, nutrients such as glucose, lipids, and amino acids which regulate the melanocortin system by affecting the activity levels of different components of the system are discussed. Among the wide range of adipokines identified over the past years and implied into physiological and metabolic control of energy balance, Bertrand et al. review the various systemic effects of apelin on energy metabolism by addressing its mechanism of action. Due to its beneficial effects on both energy metabolism and insulin sensitivity, apelin is gaining an important physiological role and is being suggested as an interesting therapeutic anti-diabetic target. Newly, in a context where no safe and effective drugs are available to treat obesity, a recent research is focused on considering the possibility that stimulating Brown Adipose Tissue (BAT) might be effective in treating obesity and its associated metabolic disorders. In a review article, Poher et al. discuss these new emerging issues and underline the perspective that new alternatives, focusing on adipose tissue function, could potentially be of therapeutic relevance in the future. A review article by Obregon examines the difference between Brown, White and Brite/Beige adipocytes underlying their contribution to energy balance supporting the reactivation of BAT and the induction of Beige/Brite adipocytes in humans as a therapeutic option to fight obesity. Moreover, this contribution reviews the role played by THs on proliferation and differentiation of adipocytes mainly via regulation of gene expression. Lecoutre and Breton review the link between nutrient supply perturbations in the fetus or neonate and increased risk of adult-onset metabolic disorders. They discuss how maternal nutritional manipulations reprogram offspring's adipose tissue predisposing offspring to fat accumulation and, thus, to metabolic disorders. The impact of adiposity on energy balance is reported in an original research article by De Andrade et al. who, by using a rat model of semistarvation-refeeding, in which fat recover is driven by suppressed thermogenesis, conclude that the putative mechanisms leading to catch up-fat involve alterations in skeletal muscle contraction, fiber composition and local TH metabolism. Another original article reports the study performed by Wang et al. who detect metabolic alterations in pre-manifest and manifest Huntington's disease patients and suggest that in addition, and prior, to overt neuronal damage, Huntington's disease affects hormone secretion and energy regulation. These findings may shed light on Huntington's pathogenesis and provide new opportunities for biomarker development. Bioenergetic aspects related to mitochondrial activity also gave a prominent contribution to this Research Topic. The physiological role of mitochondrial uncoupling proteins (UCPs) in energy metabolism is reviewed by Busiello et al. who discuss the relevance of UCPs in determining mild uncoupling, in protecting from oxidative stress and in the amelioration of fatty acids oxidation. The alterations in mitochondrial efficiency as well as the impact of this parameter on metabolic homeostasis of skeletal muscle are reviewed in the contribution by Crescenzo et al. The reported evidences led to the conclusion that an increased mitochondrial efficiency precedes and may contribute to the development of high-fat induced insulin resistance. Emerging findings reveal an important role for mitochondrial dynamics in the regulation of energy balance. Putti et al. in a review article contribution highlight the impact of diet on mitochondrial dynamic behavior and function in liver and skeletal muscle as well as the involvement of mitochondrial fusion and fission processes in body energy balance regulation in response to the nutritional status. Overall, all the original articles and the review articles covering this topic, in all their diversity, contribute to clarify unanswered questions on the physiological processes involved in the regulation of energy balance and offer clues for developing strategies to target metabolic disorders. In each of the articles contributing to this topic, there is much more than it can be commented in this short introduction. We hope that the readers will go through the articles where they will find a lot of updated information, shedding light on hormone's actions, and metabolic pathways related to energy balance which are opening new promising perspectives in the field focalized in the Research Topic.

## Author contributions

AL: contributions to the conception of the work; MM: contributions in drafting the work and to final approval of the version to be published.

### Conflict of interest statement

The authors declare that the research was conducted in the absence of any commercial or financial relationships that could be construed as a potential conflict of interest.

